# Annotations

**Published:** 1911-04-01

**Authors:** 


### ANNOTATIONS.

### Hospital Sunday: A Dangerous Policy.

We give a full report in another column of the meeting of the Council of the Hospital Sunday Fund on March 28. At that meeting a long discussion took place, and an earnest endeavour was made to find a means to secure a unanimous vote. Unfortunately, this proved impossible, and the majority of those present decided to identify the Fund with a policy which commits it to taking action in a certain event in regard to the procedure of those hospitals which run Sunday cinematograph entertainments, with the object of increasing their revenues. This decision is objectionable on three grounds. First, it commits the Council to the policy of interfering with the free action of the hospitals in selecting the sources from which to obtain necessary income. Secondly, it commits the Council of the Fund to a policy of inflicting penalties on the hospitals in certain eventualities. This action, in our view, is opposed to the best interests of the Hospital Sunday Fund, because it must tend to destroy its neutral character. There is a third and grave objection to the resolution which was adopted by a majority of the Council, for, as one speaker pointed out, the Council has no power vested in them under the Laws of the Constitution to give the instructions to the Distribution Committee provided for in the resolution without the previous consent of a meeting of the constituents of the Fund. Though the resolution states that the Council does not express any opinion upon the expediency or otherwise of Sunday entertainments, it will be seen on reference to the speeches of those speakers who supported its adoption that some of them have a very decided opinion upon this point. Looked at from every point of view the position to which the Hospital Sunday Fund has been reduced by the passing of this resolution is most regrettable, for it may be attended with results to the Fund from which we hoped it would be possible to find a way of escape. In any case the Council would be well advised to refer the legal point, upon which two conflicting legal opinions have already been obtained, to eminent counsel. We hold that the present resolution is *ultra vires*, and if so the matter will have to come before the Council again at an early meeting, and be dealt with on its merits. The Laws of the Constitution make it perfectly clear, in our judgment, that the Council of the Hospital Sunday Fund, without the previously obtained authority of the constituents of the Fund, does not at present possess the power to recommend the Committee of Distribution to take the course laid down in its resolution of March 28.

### The Sale of the Good-will of a Practice.

A curious case and one of interest to those buying and selling practices has just been decided in the French courts. A certain practitioner A transferred his practice, including the lease of a house and the names of his patients, to a doctor B, on consideration of a payment of 6,000 francs down and the promise to pay a further 4,000 francs after the lapse of a year. The time passed, but B then attempted to avoid payment of the promised sum and took the case to the courts, maintaining that the contract he had signed was not enforceable at law, seeing that the clientèle of a doctor is in its very essence a non-commercial matter, and so cannot be ceded for a money payment. The courts, however, decided against Dr. B for the following reasons: "It is decided that it can be maintained in law that the clientèle of a doctor is outside commerce and is non-existent, because it is due to the personal effort of the doctor, is maintained and increased by the professional qualities of him who created it and by the confidence he inspires in his patients; being non-existent it cannot be transmitted to a successor; but it is decided also that the clientèle was not the only object of the sale agreed to between Drs. A and B, and that the handing over of the practice was accompanied by corporeal and incorporeal elements of which the buyer could make use, notably in the taking over of the lease, the handing over of the names and addresses of patients, the agreement not to practise medicine in the neighbourhood, etc. It is decided that this last provision contains nothing that is contrary to law or that is against public order or morality, and therefore that it is capable of being the object of a contract conforming to the dispositions of Article 1126 of the Civil Code."

### Sea-water Injections.

Like all medical novelties that have obtained the advertisement of a popular notice in the Press, the treatment of certain conditions by means of injections of sea water has attracted considerable attention. We have received several inquiries as to the technique of the treatment and the possibilities of obtaining the requisite sterilised sea water in this country. Hitherto it has only been possible to arrange for a supply of injection by writing to France for it, but now Messrs. Oppenheimer and Sons, the well-known manufacturers of Bipalatinoids, have arranged to supply these injections in "aseptules" containing from 30 c.c. upwards, or in larger quantities, in sterilised bottles; and the firm will supply all information with regard to the technique. While we gladly offer this reply to correspondents, we must not be taken as advocating the treatment in any way. The exultant tone of Simons' original announcement has not been maintained in subsequent notices. In The Hospital of January 14 (p. 459) we printed an extract from a very able and full account of the treatment by Dr. De Lange, who has come to the conclusion that the treatment is scarcely worth the trouble that it entails, and that equally good results can be obtained in a less expensive manner by the employment of more conservative measures. With regard to all new methods, however, it is fair and impartial trial that is wanted, and we shall be glad to publish the accounts of such a trial if they are submitted to us in connection with the use of sub cutaneous injections of sea-water plasma.

